# Governance of China’s Potatso National Park Influenced by Local Community Participation

**DOI:** 10.3390/ijerph20010807

**Published:** 2023-01-01

**Authors:** Ting Ma, Lizhi Jia, Linsheng Zhong, Xinyu Gong, Yu Wei

**Affiliations:** 1 Key Laboratory of Regional Sustainable Development and Modelling, Institute of Geographic Sciences and Natural Resources Research, Chinese Academy of Sciences, Beijing 100101, China; 2 College of Resources and Environment, University of Chinese Academy of Sciences, Beijing 100049, China; 3 Lhasa Plateau Ecosystem Research Station, Key Laboratory of Ecosystem Network Observation and Modeling, Institute of Geographic Sciences and Natural Resources Research, Chinese Academy of Sciences, Beijing 100101, China; 4 College of Ecology, Lanzhou University, Lanzhou 730000, China; 5 Institute of Science and Development, Chinese Academy of Sciences, Beijing 100190, China

**Keywords:** national park governance, community participation mechanism, management efficiency, Potatso National Park

## Abstract

Conservationists recognize that protected areas (PAs) have limited prospects without the involvement and support of local people. As a governance strategy, community participation is to implement the coordinated development of communities and PAs. However, the effects of community participation on national park governance have rarely been tested. Therefore, the present study used a mixed-method approach that is derived from the International Union for Conservation of Nature (IUCN) green list of protected and conserved areas (PCA) conservation outcomes framework, calibrated to the indigenous peoples and local communities’ (IPLCs) self-assessments about the outcomes of community participation on national park governance to explore the community participation effects. Our results show that management efficiency controls governance outcomes. Potatso National Park’s transformation from the tourism development model to national park is still ongoing, and there exists quite a few problems. We conclude that a successful national park governance as envisaged by the “ecological civilization” paradigm requires a balance of government regulation, participation of various stakeholders in decision-making and discussion, compensation, as well as sustainable access to environmental resources by the affected populations.

## 1. Introduction

The conservation crisis, together with impoverished communities on the verges of natural PAs, poses a major challenge. The natural environment globally is losing biodiversity, highlighting the urgency of conserving PAs in order to halt this loss. Research in [1,2,3] confidently concludes that there has been an “exceptionally rapid loss of biodiversity over the last centuries, indicating that a sixth mass extinction is already under way”, with a devastating loss of ecosystem services looming [4]. The crisis, however, can be lessened through rapid intensified conservation efforts [2]. Of all the effort, PAs (protected areas) have been the most prevalent approaches shared by the governments in over 150 countries [5].

China has had high ambitions for its protected areas (PAs), largely developed following the model of strict nature reserves. In fact, China has established a vast network of nature reserves since 1956, now with 2729 nature reserves nationwide, covering about 15% of the country’s territory [6]. State-managed reserves cover two-thirds of this area [7,8]. One of the challenges inherent in nature reserves is to find ways to balance nature conservation with the needs and socioeconomic aspirations of local communities [9]. The national park model has therefore recently been introduced in China, seeking to redress previous imbalances by integrating human activities with the protection of special habitats and regulating services [10,11]. Except for national representativeness, national parks in China have two unique characteristics, which are different from those in the protected area classification system of the IUCN: ecological conservation as a first target, and public benefits for all people. China officially started the national park system pilot in 2015 and announced the establishment of the first batch of national parks at the COP15 conference in 2021. In recent years, it has been developed rapidly. Although the time is short, it has achieved remarkable results. The national park system aims, further, to advance China’s vision of “ecological civilization (shengtai wenming)” that has as goal the “harmonious development of people, nature, and society” [12]. Consequently, China is devoting more administrative resources to conservation. In some cases, the real goal is environmental protection, which means all resource exploitation has been prohibited, and this creates a predicament for the local communities. More than half of the national parks in the world overlap with the community [13]. The IPLCs in the national park have lived here for generations and have evolved into a symbiotic relationship with mutual dependence on natural resources. The establishment and construction of national park have caused the IPLCs to suffer from the loss of livelihood dependence, the obstruction of livelihood methods, and the limitation of livelihood scope, resulting in fierce space competition and conflicts of interest between communities and national parks [14].

The IPLCs are an important component of the ecological elements of the national park [15]. How to reconcile the conflict between the ecological protection of national parks and the protection of IPLCs’ rights and interests has become a common dilemma in current national park governance [16]. Community participation in national parks is a positive way to resolve conflicts, share benefits, and improve the effectiveness of park protection [17,18]. In the “Overall Plan” of China’s national parks, it is clearly proposed to build a “community participation mechanism” suitable for national and local conditions to coordinate or avoid conflicts between the needs of national park ecological protection and the demands of indigenous peoples’ development. Completion of community participation directly determines the sustainability of national park management [19,20]. The mechanism of community participation in national parks originated in the United States in the 1960s and 1970s [21] as an autonomous management policy that includes IPLCs. After a long period of evolution [22,23], it has become one of the most crucial components of the management measures of PAs.

Many studies focused on the importance and the influencing factors of community participation; the majority of research has focused on the macro level [24,25,26]. In particular, Khwaja [27] examined the impact of community participation on development project outcomes and found that community participation may not always be desirable. In practice, the shortage of professionals limits the form and extent of community participation in protected area management. Marcus [28] pointed out that most of the “participatory” projects are initiated and directed by outsiders, the time is very short, and they focus on the trial and practice of technology, failing to achieve sustainable development. Good community participation practices are creative attempts by local communities to solve problems they face. China has carried out national park construction and some scholars have conducted research on community participation in management [29], such as the specific promotion process and case studies of the community participation of typical national parks in the United Kingdom, France, and Australia have been introduced. However, due to differences in politics, social and cultural customs, as well as the population density of PAs, land ownership, and the roles and strengths of the communities themselves, the advanced practical experience of other countries is not fully applicable to the management of China’s national parks. The national park community in China is the largest in the world. Due to its wide spatial distribution, complex and diverse types, and huge cultural differences, the community participation of national parks in China is also the most complex and challenging in the world. If an appropriate and effective community participation mechanism cannot be created and selected according to the national conditions, the sustainable development of China’s national parks cannot be guaranteed at all. Overall, the research and practice of community participation in national parks in China lacks systematic exploration of governance outcomes; again, it confuses the concepts of community participation in nature reserves and national parks. In short, the empirical research to explore the community participation’s outcomes and mechanism in national park governance still needs to be improved.

The contribution of community participation for biodiversity conservation and socioeconomic development is not well determined in China’s PAs as a whole and particularly in the northwest Yunnan region, hence why this study was initiated. In addition, under the guidance of national park governance goals (China’s national park governance has multiple goals of biodiversity conservation, ecosystem function improvement, and win–win outcomes for all stakeholders, and the goal of community participation is to achieve the coordination of ecological protection and community interests), due to historical and realistic factors, sometimes the behavior of community participation under the concept of community governance is oriented incorrectly, yet process feedback can guide the direction of future governance. Whatever the policy and/or research processes are followed, and challenges are encountered, learning from experiences and channeling key lessons learned into future development programs and actions are of paramount value.

The significance of our paper is to contribute to ensuring that the development of the current approach used by the government of China (i.e., the development of a national park system) is carried out in ways that are effective for local communities, since, based on global experience, failure here would likely also lead to failure in attaining the desired longer-term conservation outcomes. Therefore, this research ensures the participation level of the local community in the implementation of the national park system, as they are dependent on the natural resources, and their involvement is crucially needed to bring social and environmental co-benefits.

## 2. Materials and Methods

### 2.1. Survey Areas

Potatso National Park (also named as Shangri-la Potatso National Park) System Pilot Area is located in the Hengduan Mountains in Southwest China. It lies in Shangri-la City, Diqing Tibetan Autonomous Prefecture, Yunnan Province [30]. It is the core area of the Three Parallel Rivers World Natural Heritage site, a national key functional area for biodiversity protection and water conservation in the Northwest Plateau of Yunnan Province, and also a region where the three globally major biodiversity hotspots gather in the world. It is regarded as a world-class gene bank of species [31,32,33]. Although the biodiversity and ecological environment of northwest Yunnan are rich, they are very fragile. At the same time, northwest Yunnan is a typical remote area inhabited by ethnic minorities. While the protection is continuously strengthened, it also limits the traditional livelihood of the local people and the economic development of the region [34].

The rich but fragile ecological environment in northwestern Yunnan urgently needs to be protected; meanwhile, the economic and social development of Tibetan areas also should be accelerated [35,36]. This is especially true after the natural forest protection project’s comprehensive implementation in northwestern Yunnan in 1999; the local fiscal revenues that used to rely mainly on deforestation have been prohibited. Then, the IPLCs’ livelihood was seriously affected, so they entered into the Bitahai Provincial Nature Reserve to develop eco-tourism to make a living.

To effectively conserve the world heritage sites, and to ensure the IPLCs’ sustainable development, a new PA model that enhances both ecosystem health, through conservation, and local livelihoods is urgently needed [37]. In 2006, the Yunnan Provincial People’s Government explored a win–win path for protection and development based on the northwest Yunnan region’s situation and took the lead in establishing the first national park in the mainland—Shangri-La Potatso National Park—which was listed as the State Forestry Administration’s pilot project in 2008. In 2015, it was listed as one of China’s ten national park system pilots [38].

**Figure 1:**
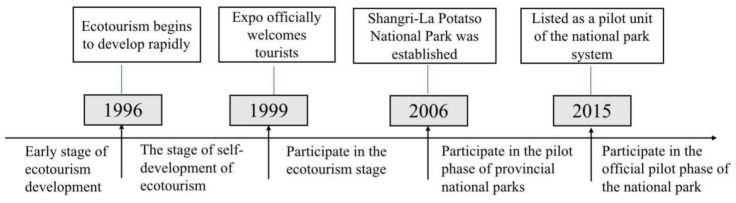
Potatso National Park Community Development History.

Since 2006, the national park carried out “Potatso National Park Tourism Feedback Community Implementation Plan” that involved 2 townships (Jiantang Township and Luoji Township), 3 village committees (Hongpo Village, Jiulong Village and Niru Village), 23 villager groups, and 870 households. Potatso National Park mainly focused on the IPLCs who were engaged in horse-raising activities in the former Bitahai West Line, South Line and Shudu Lake before 2006, also including those engaged in business activities, such as barbecues, photos, and clothing rentals in the national park [39]. For protecting the ecological environment and regulating the disorderly business activities, those who withdrew from horse-drawing and other business activities were compensated. The years from 2008 to 2012 were the first five years for the national park to carry out its first-round community back-feeding program, and the second round was from 2013 to 2018. The scope is divided into three categories: first-, second-, and third-class area for compensation with different standards. The back-feeding work mainly included the following aspects: First, it was implemented by direct cash compensation according to the household’s income. Second was to subsidize the first-class area children’s education. The third was to set up community public welfare posts. The fourth was to invest in and build community infrastructure. Potatso National Park has explored a win–win path to effectively protect biodiversity and coordinate economic and social development [40]. However, the community back-feeding funds’ source is single, which still relies on the national park’s tickets income to provide “blood transfusion” compensation [33]. Potatso National Park’s community development has undergone five stages (see Figure 1), including the ecotourism’s pre-development, the ecotourism’s self-development, participation in ecotourism stage, participating in the provincial national park pilot stage, and participating in the national park pilot stage.

### 2.2. Methods

#### 2.2.1. Conceptual Framework and Research Hypotheses

**Figure 2:**
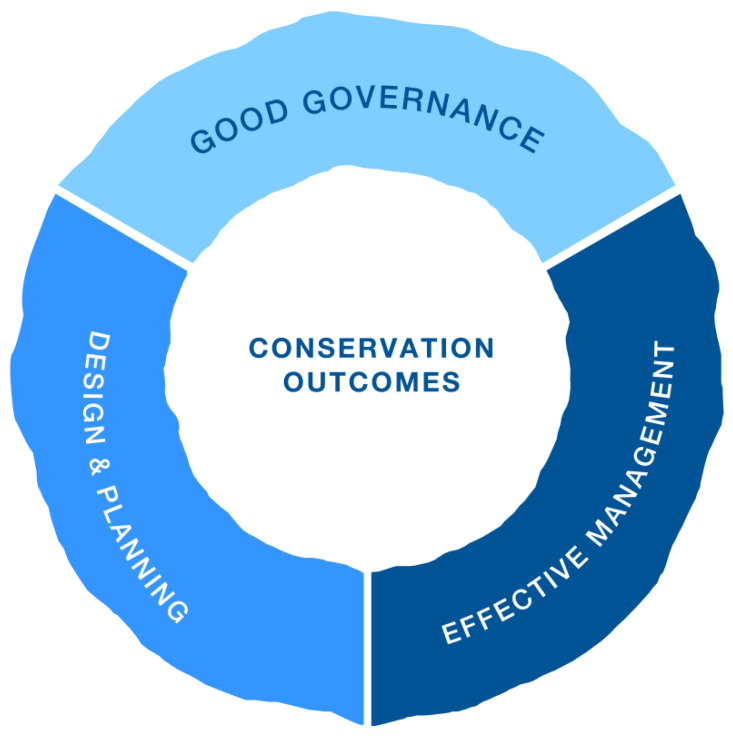
The IUCN Green List of Protected and Conserved Areas (PCA): providing globally consistent, locally relevant standards. Adapted from Hockings et al. (2019) [49].

**Figure 3:**
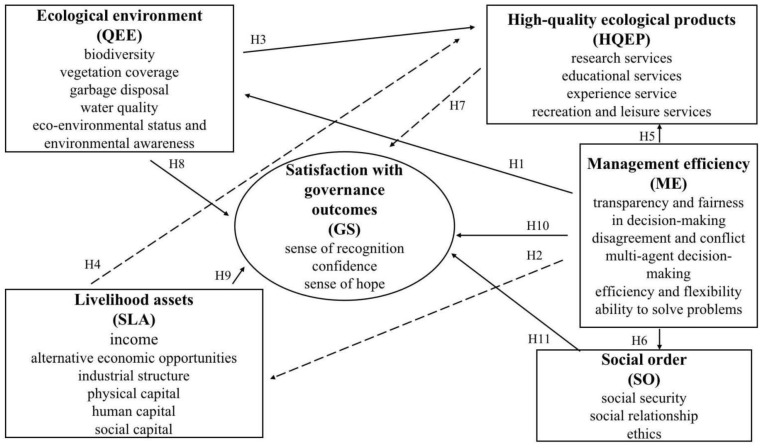
Proposed structural model.

Here, we followed a specific version based on the above-mentioned IUCN green list of PCA conservation outcomes framework (See Figure 2 and Figure 3), the following key thematic areas were covered in our new version (not only refers to the core content of the IUCN green list of PCA conservation outcomes framework, but also adds various aspects of the functions of China’s national parks), as displayed in Figure 2 to depict heterogenous interactions of IPLCs’ perception [41] about ecological environment [42,43], high-quality ecological products [44], management efficiency [45], social order [46], livelihood assets [47], and satisfaction with governance outcomes [48] in the Potatso National Park context (Table 1). Now, considering that the goal of our research was to better understand the governance and management of the Potatso National Park, within the Potatso National Park regional landscape, identification of the factors contributing to governance outcomes influenced by local community participation were the primary goals or purposes of this research, noticing, especially, that governance outcomes are achieved through a combination of the quality of ecological environment, status of livelihood assets, and high-quality ecological products, management efficiency (these four aspects are the standards for measuring the functions realization of the national park), social order, and satisfaction with community participation.

**H1.**  *Management efficiency positively affect the attitude of IPLCs toward ecological environment.*

**H2.**  *Management efficiency positively affect the attitude of IPLCs toward livelihood assets.*

**H3.**  *Ecological environment positively affects high-quality ecological products.*

**H4.**  *Livelihood assets positively affect the high-quality ecological products.*

**H5.**  *Management efficiency positively affect the high-quality ecological products.*

**H6.**  *The attitude toward management efficiency positively affects the social order.*

**H7.**  *High-quality ecological products positively affect the satisfaction.*

**H8.**  *Ecological environment positively affects the satisfaction with governance outcomes.*

**H9.**  *Livelihood assets positively affects the satisfaction with governance outcomes.*

**H10.**  *Management efficiency positively affect the satisfaction with governance outcomes.*

**H11.**  *Social order positively affects the satisfaction with governance outcomes.*

#### 2.2.2. Procedure and Sample

The data in the study were obtained by a household survey. First, interviews were guided by the staff of the Potatso National Park management department, village heads, and 5 local residents were contacted to elicit readily accessible data on ecological environment, livelihood assets, high-quality ecological products, management efficiency, social order, and satisfaction. In these interviews, three open-ended questions were employed: “What benefits or harms do you think the community participation in governance of Potatso National Park?”; “Which individuals or organizations may influence the assumption of governance effect?”; and “What factors may promote or obstruct the governance effect?”. Afterward, a pilot questionnaire on the governance outcomes of community participation in Potatso National Park was developed based on our newly derived framework (based on SES and IUCN frameworks), former interview results, and consultation with experts [50]. A total of 50 questionnaires were obtained in the pilot survey, and the data were used to select reliable and valid items and improve the quality of the formal questionnaire. In the third step, a standard questionnaire was constructed on the basis of the results of the pilot questionnaire survey. Some unreliable and invalid items were excluded to improve the internal consistency and discriminant validity. The final version of the questionnaire comprised two parts: the demographic and socioeconomic information of the participants and their families and the participants’ agreement and responses to the related questions. A five-point Likert scale was used to evaluate each topic.

The formal survey was conducted by the senior author and a Tibetan–Mandarin translator from March 2022 to April 2022 in the format of face-to-face interviews in the participants’ houses. A disproportionate stratified sampling method was used to choose the respondents. The first-class communities mainly involved in Potatso National Park include the Luorong Community of the Hongpo Village Committee of Jiantang Town, with 33 households and 164 local residents, and the Jilu Community, Xialang Community, and Cichiding Community, with 75 households and 375 local residents. The second-class communities include the Jiulong Village Committee of Luoji Township (including the resettlement group), with 335 households and 1,173 local residents; the Second Community of Hongpo Village Committee of Jiantang Town, with 110 households and 559 local residents; there are 186 households and 487 local residents in the Sanshe of Hongpo Village Committee of Jiantang Town. The third-class communities include the Niru Village Committee of Luoji Township, with 119 households and 650 local residents. There is a settlement of Luorongshe in Potatso National Park with 33 households. There are two townships around Potatso, Jiulong Village inhabited by the Yi people, and Luoji is inhabited by the Han and Naxi people, and the rest are inhabited by the Tibetans, who are mainly semi-agricultural and semi-pastoral.

Based on the principles of feasibility and representativeness, all three types of communities were selected for this research, 399 local residents were interviewed during the limited survey time. Information was provided by the head of each household. The government translator played only the translation role, and we attempted to stratify the sample to represent different types of households or household heads (e.g., by age, gender). A total of 399 household representatives were interviewed, and their responses were recorded both by hand and by audio recorder. After transcribing all the responses to the datasheet, 382 local families interviewed with the complete set of responses comprised the study group of this study.

#### 2.2.3. Modeling

Cronbach’s alpha coefficient was used to test the reliability of the data, and confirmatory factor analysis (CFA) was adopted to test the validity of the data. After the reliability and validity tests (see Table S1), a structural equation model (SEM) was used to measure the proposed theoretical model and test the research hypothesis. SEM has been widely used to examine the relationship between multiple independent variables and dependent variables. It includes measurement and structural models. The measurement model examines the relationship between the latent variables and the observed measures, whereas the structural model explains the structural relationship between the latent variables [51]. In this study, SPSS 24.0 software were used for data processing, analysis, and model fitting.

## 3. Results 

### 3.1. Descriptive Information of Samples

We collected data from 382 validated questionnaires. Of the 382 respondents, 134 were women and 248 were men. Most respondents were aged 35 to 45 (*n* = 195, 51.05%) and 20 to 35 (*n* = 95, 24.87%). Most respondents have college (*n* = 158, 41.36%) education. The annual income of most respondents is less than CNY 50 thousand (USD 7.16 thousand in 2022) (*n* = 274, 71.73%). The overall income level of the sampled IPLCs is relatively low, which is in line with the actual situation of rural households in Yunnan Province. Detailed demographic information is shown in Table 2.

### 3.2. Exploratory Factor Analysis

The EFA and reliability testing results of the formal survey are shown in Table S2. For each construct of the model, the standard factor loading of each observational variable was >0.70 and the Cronbach’s α value was >0.60, indicating that the latent variables can be explained well by their relevant observable variables. The Cronbach’s α value was 0.94 for the hypothesis model, which shows that the model had a high degree of reliability and it was sufficiently reliable for analysis. In addition, the parameter KMO was 0.93 and the Bartlett test of sphericity *p* < 0.001 proved that the data were suitable for factor analysis.

### 3.3. Model Analysis

**Figure 4:**
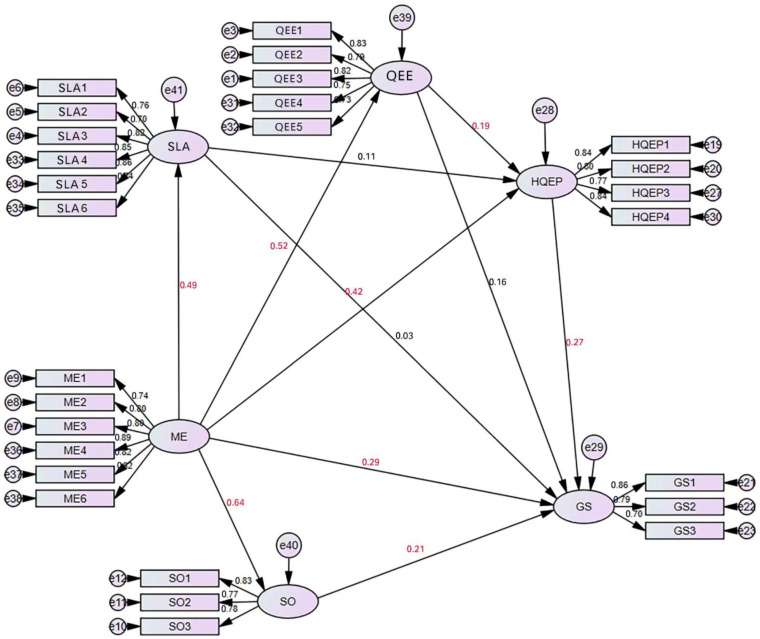
Standardized path coefficients of the final structural model for our new framework: arrows in black mean significant effect while those in gray indicate insignificant effect.

The standardized path coefficients of the structural model for the new framework are shown in Figure 4. Ecological environment, high-quality ecological products, management efficiency, and social order were all positive in predicting satisfaction. The most important decisive factor of satisfaction was management efficiency, with a path coefficient of 0.29 (*p* < 0.05), followed by high-quality ecological products with a path coefficient of 0.27 (*p* < 0.05). This indicates that IPLCs’ satisfaction with community participation in national park governance mainly depends on management efficiency. Ecological environment had the smallest impact on satisfaction, as its path coefficient was 0.16 (*p* < 0.05). In addition, management efficiency was positively related to ecological environment, with the path coefficients of 0.52 (*p* < 0.05).

There was no significant relationship between livelihood assets and satisfaction. Furthermore, quality ecological products were positively affected by the ecological environment with a path coefficient of 0.19 (*p* less than 0.05). However, there was no significant relationship between livelihood assets and quality ecological products (path coefficient = 0.11, *p* > 0.05). Management efficiency was positive to social order, livelihood assets, and high-quality ecological products, with path coefficients of 0.64 (*p* < 0.05), 0.49 (*p* < 0.05), and 0.42 (*p* < 0.05), respectively.

### 3.4. Hypothesis Testing

The results of the hypothesis test shown in Table S3 indicate that H1, H2, H3, H5, H6, H7, H10, and H11 were completely adopted and H4, H8, and H9 were rejected.

## 4. Discussions 

### 4.1. Use the New Framework to Explore the Outcomes of Community Participation on National Park Governance

This study validates the applicability of using an innovative framework to analyze IPLCs’ perceptions and attitudes about the effectiveness of community participation in PA governance. Exploratory factor analysis and full-scale reliability test results show that the analytical framework is appropriate. The SEM analysis shows that the interrelationships among ecological environment, livelihood assets, high-quality ecological products, management efficiency, social order, and satisfaction are well revealed (See Section 3.2 and Section 3.3). PAs are often surrounded by impoverished communities. Biodiversity must be conserved while improving community wellbeing [52,53]. Greater insight is required into what influences pro-conservation attitudes and behavior in these communities. Much appears to rest on the relationships between protected area staff and local communities surrounding the parks [54], yet there is limited understanding of stakeholders’ perceptions and how to pragmatically achieve win–win solutions [55,56]. With the current lack of a multidimensional framework to enhance understanding of these complex relationships, this research aimed to construct a comprehensive integrated framework representing the components that can influence people–park relationships [57]. Therefore, by providing a robust new model for the practice of community participation on PAs’ governance outcomes, this study addresses concerns about the lack of a suitable framework to explore how IPLCs fulfill PAs’ functions in the governance process. This study highly supports the hypothesis that management efficiency affects the outcomes of IPLCs’ participation in PA governance (Table S3). The efficient management efficiency of the local government has a more prominent effect on community participation. Similarly, Franco [58] reported that efficient management efficiency of local governments has a significant positive impact on PA governance. Therefore, it is appropriate to use management efficiency to measure the outcomes of PA governance. In this study, management efficiency was identified as the first important factor influencing the outcomes of community participation on protected area governance (see Section 3.3). This finding is consistent with Dawson [59]. Therefore, the management efficiency of Potatso National Park should be strengthened.

### 4.2. Effect of Farmers’ Livelihood Assets on Quality Ecological Products and Their Satisfaction

The results showed that livelihood assets had no positive effects on satisfaction (Table S3), which was consistent with the study of Ma et al. [5]. This was also confirmed in the natural resource management study in Cambodia [60]. Although the degree of influence of attitudes on community participation varies spatially, the importance of attitudes on community participation has been consistently confirmed in Thailand [61] and Ethiopia [62]. Additionally, the respondents mentioned several times that the back-feeding agreements are thoughtful for IPLCs, and the average household can receive back-feeding income ranging from tens of thousands of yuan (CNY) per year in our survey. However, its implementation is not ideal. Since 2018, the Potatso Tourism Branch has not honored the feedback agreement from the IPLCs. Potatso Tourism Branch is the abbreviation of Potatso Tourism Branch of Diqing Prefecture Tourism Group Co., Ltd., which was established on 24 July 2007 by Diqing Prefecture Tourism Development Group in Potatso National Park. According to the local government No. 20 document [2004]’s content, the company is solely responsible for the national park’s assets, operation, and management rights, including the construction of scenic spots. Up to now, the total assets of the company are nearly CNY 346 million (USD 49.54 million in 2022), the total liabilities are nearly CNY 186 million (USD 26.63 million in 2022), and the asset-liability ratio is 53.75%. There are 342 employees and 95 operating vehicles, as well as 8 cruise ships participating in tourism services [63]. This, coupled with the community lacking opportunities to participate in the national park’s co-governance, has meant that the issue regarding the community back-feeding is still the contradictions’ focus [40]. Since Potatso National Park is changing from a tourism development model to national park model, the key conceptual change is from the original development priority to the ecological protection firstly. Therefore, IPLCs in the national park have to give up some previous horse-drawing and grazing activities that affect environmental protection. The Potatso Tourism Branch’s failure to fulfill the back-feeding agreement and the IPLCs livelihoods’ reduction explained the result that livelihood assets did not have positive effects on satisfaction.

In addition, we also ask, “Is imported currency a sustainable way of making a living?”. It may be useful to consider different types of motivations and how they are affected by policy [64]. Tourism back-feeding is a form of “extrinsic” motivation, while people also have strong “intrinsic” motivations, they do things because of their own values or what brings them real enjoyment. Some intrinsic motivations may be related to culture and tradition [2]. The government hopes to replace intrinsic motivation with extrinsic motivation, which may be possible to a certain extent, but governments should also consider how to maintain and “squeeze in” intrinsic motivation [31].

The local government’s governance policies or plans did not consider, in the design of Potatso National Park, the intrinsic motivation of community participation. Now, the IPLCs are not enthusiastic enough to participate in the national park’s co-construction. The reason is that the national park’s operation and management was not directly related to IPLCs, which led IPLCs to deem that as long as they do not participate in the ecological environment’s destruction, the national park’s construction has nothing to do with them. Absence of a substantive element of community participation in the national park system results in a lack of ownership by IPLCs [40]. Therefore, attention should be paid to enhancing IPLCs’ enthusiasm for environmental protection and willingness to supervise the environment during the process of implementing policies or plans in the future [65]. The compensation agreement method after 2018 may be guaranteed to the national and provincial governments in the form of ecological compensation on the basis of striving to establish a diversified financial guarantee system, which seems a long-term solution. In addition, providing scientifically appropriate franchise projects to increase the IPLCs’ income is also an effective way to improve the national park governance’s effectiveness. It is worth noting that the squeeze-in of intrinsic motivation is also crucial, given that livelihood assets did not show a significant impact relationship on high-quality ecological products, which was also confirmed in the study of Lan et al. [66]. During the interview and field investigation, it was found that Potatso National Park lacks high-quality ecological products. According to the available information and the results of on-site interviews, it was found that the national park does not pay much attention to the preservation of the community ethnic culture. Actually, the unique ethnic culture is the Potatso National Park development’s potential [36], yet it is lagging behind in exploration and protection, as well as the utilization of community cultural resources. 

The excavation of high-quality ecological products that highlight the characteristics of Potatso National Park with culture as the source is suggested on the basis of conserving community cultural resources [36]. Due to the limited education level of IPLCs, they do not understand the concept and definition of high-quality ecological products. The survey also found that the IPLCs’ “cognitive stress” on “Potatso National Park” falls on the word “park”, that is, most local villagers do not fully understand the connotation and concept of “national park”, but only think that Potatso is a park or a national scenic spot. Therefore, Potatso National Park’s co-construction calls for complete understandings about the concept and definition related to national parks through relative publicity and education for guiding IPLCs’ future behavior.

The analysis of the community participation’s dilemma in Potatso National Park showed that its hidden deep reason lies in a lack of community subjectivity and sense of belonging in the national parks’ co-construction [67,68]. Early studies have mainly focused on the influences of cognitive factors (such as knowledge level and economic rationality) on pro-environmental behavior, whereas the role of emotional factors has been usually ignored [69]. Almeida et al. [70] found that no obvious correlation exists between environmental cognition level and pro-environmental behavior. Recently, many scholars have begun to analyze the effects of cognitive and emotional factors on pro-environmental behavior and found that the latter has significantly higher explanatory power than that of the former [71]. As a hot psychological concept in community research, sense of belonging is also one of the three elements of the basic psychology needs theory [72]. According to Maslow’s hierarchical theory of needs, the need to belong refers to individuals’ desire to join a certain organization or group and be accepted and respected by group members [73]. Thus, the sense of belonging refers to individuals’ emotional connection to the place and the group of people in which they live [74]. For IPLCs, the sense of belonging is the villagers’ psychological feelings of identification, affection, and attachment to the village they live in and the surrounding people [75]. Considering the emotional bond between IPLCs and the national park, their sense of belonging helps national parks to promote participation in environmental governance more actively. Studies have shown that farmers with a higher sense of belonging are more willing to prioritize collective interests over personal interests and take the initiative to undertake more responsibilities and obligations to seek the long-term development of the collective [76]. Sense of belonging can motivate IPLCs to establish behavioral goals of national park environmental protection. According to the viewpoint of human geography, people’s behavior toward resources and environment is influenced by a “human–land” relationship formed by place attachment [77]. Place attachment and place identity can promote IPLCs to show pro-environmental behavior for environmental protection [78,79]. In the future, more work needs to be carried out on the enhancement of IPLCs community subjectivity and sense of belonging.

### 4.3. Mutual Relations between Satisfaction, Ecological Environment, Livelihood Assets, High-Quality Ecological Products, Social Order, and Management Efficiency

The results show that management efficiency has a significant positive relationship with satisfaction, ecological environment, livelihood assets, high-quality ecological products, and social order. These results were also observed in previous research on community participation in natural resource management [80]. However, compared with others, the path coefficient of management efficiency to satisfaction is the lowest (see Table S3). The explanation for this is due to the narrow scope of community participation. At present, the way for the community to participate in the national park co-construction is to provide tourism feedback for IPLCs based on the park’s use of community resources. The community fails to actively participate in the construction, operation, management, and development of the park, resulting in no relationship between the community and the park except for the intersection of tourism and feedback. The national park lacks long-term planning for the path of community development paths and fails to play an active role in coordinated development with surrounding communities. This finding is also consistent with Maslow’s hierarchy of needs theory [73]. Therefore, improving the living conditions of IPLCs is a prerequisite for improving their attitudes towards the community participation mechanism.

The standardized path coefficient of management efficiency to social order was the highest (0.64). It shows that the positive effect of management efficiency on social order is significant. In future work, attention should be paid to improving the enthusiasm of IPLCs to participate in the co-construction of the park. To fully understand the new ecological protection concept of the national park, it is important to change “passive” protection to “active” participation, improve the sense of ownership, guide IPLCs to fully realize the importance of coordinated development of national parks and communities, and effectively participate in the whole process of national park construction. It is also important to increase gratitude education for IPLCs so that they can fully realize the superiority of the tourism feedback policy, consciously practice their own obligations and responsibilities, and fundamentally solve the ideological problems of IPLCs.

The standardized path coefficient of management efficiency on the ecological environment is 0.52, and the *p* value is less than 0.05, indicating that there is a significant positive impact relationship, which is consistent with the results that Rehman et al. [81] observed. The main purpose of establishing national parks in China is to protect nationally representative natural ecosystems. Therefore, the management department has always adhered to ecological environmental protection and put ecosystem protection first in the formulation and implementation of relevant management policies. With the improvement of management efficiency, the ecological environment also tends to improve. It is worth noting that there is an ongoing conflict between the livelihood development of the national park IPLCs and ecological protection. The biodiversity and poverty crises are realities and impact each other. In the African context, protected area management confronts very real constraints in terms of budgets and a limited number of staff, who need to accomplish a multitude of tasks. Communities bordering these parks are often impoverished and may not share the sentiment that these parks should be protected. The conservation of this crucial biodiversity thus depends on good relationships with surrounding communities and finding win–win solutions.

The standardized path coefficient of management efficiency on livelihood assets is 0.49, and the *p* value is less than 0.05, indicating that there is a significant positive impact relationship, and the hypothesis is established. The establishment of China’s national parks emphasizes the public welfare of the whole people. In the case of IPLCs, as one of the main stakeholders, ensuring their livelihoods’ sustainability is the national park construction’s responsibility. Compared with other countries, the IPLCs living in or around the China’s national parks are often economically underdeveloped, the conflicts between IPLCs’ livelihoods and the environmental protection never go away. The Potatso National Park is located in Yunnan Province, IPLCs have certain livelihood vulnerabilities. Therefore, a series of ecological compensation measures for IPLCs were provided when the national park was established, such as the tourism back-feeding agreements and job creation, etc.; multiple measures have improved the IPLCs’ livelihood assets. However, what follows is the IPLCs’ dependence on back-feeding agreements [82]. Future management should improve IPLCs’ livelihood ability and reduce their dependence on compensation.

Although China recognizes the importance of community participation in its new national park system in northwest Yunnan, most actual decision-making (i.e., governance) [83] in China’s PAs is still dominated by top-down habits, further accentuated by the continued “belief” in the unquestioned efficacy of rational-comprehensive planning theory “with its roots in economic assumptions about human behavior [84] and positivist assumptions about science [85] [often leading to exclusive] application of the scientific method to decision-making” [86]. This theory holds the (erroneous) view that planners can be “value-neutral technicians” who undertake purely objective analyses of planning problems. However, such approaches do not work well—if at all—with complex or wicked problems. Accordingly, a rethinking of problems from the perspective of a social–ecological systems framework creates new opportunities for development, in particular through “adaptive governance” that includes “the pursuit of adaptive and integrated goals, utilization of diverse sources of knowledge, reliance on a polycentric institutional structure, and an analytic-deliberation decision-making process that is informed by communicative action theory” [86,87,88,89]. Most significantly, as begun here in this study—and made possible through community participation (although there are gaps with what is observed elsewhere in the world) that is already being trialed in the Potatso National Park—it is essential to recognize and listen to local voices, with their local perspectives; mutual understanding across stakeholder groups, integrating multiple ways of knowing, and co-creating solutions are all essential for developing viable models of regional conservation and development [90,91].

### 4.4. Governance Recommendations

#### 4.4.1. Enhancing Community Participation’s Ability

It is necessary to fully understand the close relationship between the community and the Potatso National Park’s ecological environment and cultivate more qualified IPLCs to participate in the national park’s management [58,59]. It is recommended to learn from the Sanjiangyuan National Park co-management mechanism and other PAs’ experience. For example, in South Asia [92], to combat the continuous deterioration of forest resources, community co-management has been introduced as a permanent approach to integrating local communities in forest management processes by utilizing the capacities and comparative advantages of various social actors. This approach seeks to enhance both forest health through conservation, and local livelihoods by offering local communities the responsibility to manage forest resources alongside the opportunity to enjoy benefits derived from them. Co-management approaches are widely considered to be a successful natural resources conservation technique to address anthropogenic disturbances in many regions of the world, such as Nepal, Honduras, Ethiopia, and Malawi [93,94,95,96].

#### 4.4.2. Reimaging the Community Franchise Mechanism

In view of the current situation that Potatso National Park did not have a normative franchise mechanism, it is recommended to propose standards as soon as possible. It is recommended to comprehensively consider all business activity characteristics, and then create franchise models in the national park’s context, so as to avoid the situation that the national park management agency is both an athlete and a referee [97]. The second is to reduce national park admission prices to reflect the national park’s public welfare. Learning from the fact that the operation of American national park systems was mainly supported by federal finances, then admission fees, franchise revenue, and social donations are only supplements. Noticing that the multi-day admission fees are only around USD 10, which fully reflected the national park’s public welfare. We should have a correct understanding that public welfare is at the national park system’s core. Access to national parks should become a basic public service provided by local government, allowing all individuals to form a so-called national pride through barrier-free experiences of cultural and natural heritage sites [98].

### 4.5. Limitations of this Study

This study provides an important insight into the use of adapted IUCN green list of PCA conservation outcomes framework to explore factors affecting IPLCs’ intention toward governance outcomes in the national park. Even so, there are some aspects that need to be improved in future studies. The latent variables used were difficult to measure entirely and accurately. Bergen and Latkin [99,100] reported that respondents tend to answer questions in a manner that will be viewed favorably by others. The IPLCs’ positive feelings may be exaggerated because they may have answered the questions according to what they think the researchers would want to hear [101]. This occurrence may lead to a more conspicuous social desirability bias and, eventually, biased data. This issue is inherent to social surveys [102,103]. A number of countermeasures were taken to reduce this bias, including providing more detailed explanations prior to the questionnaire items and encouraging IPLCs to be honest.

## 5. Conclusions

This study explored the community participation in the construction of Potatso National Park by means of a mixed-method approach that is derived from the IUCN green list of PCA conservation outcomes framework, calibrated to IPLCs’ self-assessments about the outcomes of community participation on national park governance. The results show that the ecological environment has a positive impact on high-quality ecological products; on the contrary, livelihood assets are not significant. In addition, management efficiency has a significant positive impact on the ecological environment, livelihood assets, high-quality ecological products, and social order. Overall, this study expands the IUCN green list of PCA conservation outcomes framework to identify the driving factors of influencing community participation outcomes on national park governance, thereby unlocking opportunities for community management in China’s PAs and providing a theoretical basis for management practices in other areas. We suggest mainstreaming community participation effects in national park management in China and elsewhere. The community participation system should also be more dynamic by incorporating appropriate patterns and paths that are more adaptive to the community participation paradigm guided by national park governance, considering and strengthening IPLCs’ adaptive capacity and resilience to achieve the function of the national park.

## Figures and Tables

**Table 1:** Constructs and measurements of the hypothesis model.

Constructs	Variables	Description of Question
QEE	QEE1	Biodiversity has been protected (wild animal populations and numbers have increased).
	QEE2	Vegetation coverage has increased (including grasslands, meadows, trees, etc.).
	QEE3	Disposal of waste in the community has been improved.
	QEE4	The water quality is better than before.
	QEE5	The surrounding natural environment has improved, IPLCs’ awareness of environmental protection has been improved, and ecological environment damage such as tree cutting and poaching has been significantly reduced.
SLA	SLA1	After the community participated in the governance, the household income of the IPLCs increased significantly.
	SLA2	Alternative economic opportunities for communities have increased, promoting sustainable economic development in the region (increased employment opportunities) increase, receive sustainable compensation, etc.).
	SLA3	The industrial structure of the community has been improved, and the proportion of the tertiary industry has increased.
	SLA4	The physical capital of the community has been improved (including the community’s transportation, housing, road transportation, water, electricity, etc., infrastructure).
	SLA5	Human capital has been improved (e.g., IPLCs—enhanced production skills; forest rangers—enhanced protection, identification, monitoring capabilities; township managers—more professional and enhanced management capabilities).
	SLA6	Improved social capital (promoting trust, cooperation, and mutual understanding among IPLCs, NGOs, and township government staff).
HQEP	HQEP1	Scientific research services in national parks have been enhanced.
	HQEP2	Educational services in national parks have been enhanced.
	HQEP3	Experience services at national parks have been enhanced.
	HQEP4	Recreation and leisure services in national parks have been improved.
ME	ME1	Decision-making is more transparent and fairer.
	ME2	Differences and conflicts between actors with different interests and ideas have been effectively resolved.
	ME3	Community participation provides greater support for management of decision-making and truly realizes multi-agent decision-making.
	ME4	The management team has greater efficiency and flexibility in making decisions and responding to problems.
	ME5	The ability to solve problems has improved, and it has been able to expand the scope of management and meet greater challenges.
	ME6	A protocol for solving practical problems was produced.
SO	SO1	Social security has been improved.
	SO2	Social relations (villager family relations, mutual relations between villagers, relations between villagers and village cadres, and relations between villagers and migrant households) have been improved.
	SO3	Ethics are more standardized.
SWGO	GS1	You agree with the current effectiveness of community participation in governance and are satisfied with the achievement of governance and management goals.
	GS2	You have confidence that community governance will continue to successfully achieve its goals and will continue to support its development.
	GS3	You are hopeful about the future prospects of community engagement in governance.

**Table 2:** Respondent profile (*n* = 382).

Social Attribute Characteristics		Samples Frequency	Proportion (%)
Gender	Male	248	64.92
	Female	134	35.08
Age	20–35	95	24.87
	35–45	195	51.05
	45–60	90	23.56
	61 and above	2	0.52
Ethnic	Tibetan	207	54.19
	Han nationality	59	15.45
	Hui nationality	11	2.88
	other	105	27.49
Education level	Not been to school	9	2.36
	Primary school	38	9.95
	Junior high school	70	18.33
	Senior high school	30	7.85
	college	158	41.36
	University and above	77	20.16
Personal annual income (CNY)	Below 5,0000	274	71.73
	5,0000–10,0000	87	22.78
	10,0000–20,0000	16	4.19
	20,0000–30,0000	3	0.79
	Above 30,0000	2	0.52

## Data Availability

Our specific datasets generated in this study are not publicly available, as they are part of the authors’ ongoing research. These data are available from the corresponding authors upon reasonable request.

## Supplementary Materials

The following supporting information can be downloaded at: https://www.mdpi.com/article/10.3390/ijerph20010807/s1, Table S1: Reliability Statistics; Table S2: Goodness of fit measures of SEM model; Table S3: Hypothesis testing results.
