# Supplementary material for: Governance of China’s Potatso National Park Influenced by Local Community Participation
Source: Int J Environ Res Public Health. 2023 Jan 1;20(1):807. doi: 10.3390/ijerph20010807 (PMC9819458; doi:10.3390/ijerph20010807)
Supplement: Supplementary file 1 [file ijerph-20-00807-s001.zip › ijerph-2095843-supplementary.pdf]

**Table S1.** Reliability Statistics.

| Variables | Cronbach Alpha | Number of Items |
|---|---|---|
| Ecological environment | 0.887 | 5 |
| Livelihood assets | 0.905 | 6 |
| High-quality ecological products | 0.884 | 4 |
| Social order | 0.835 | 3 |
| Management efficiency | 0.921 | 6 |
| Satisfaction with community participation | 0.823 | 3 |
| Overall Cronbach's alpha value | 0.939 | 27 |
| Kaiser–Meyer–Olkin | 0.934 | |
| Bartlett test of sphericity | <0.001 | |

**Table S2.** Goodness of fit measures of SEM model.

| Index | $X^2$/df | GFI | RMSEA | CFI | NFI | NNFI |
|---|---|---|---|---|---|---|
| Recommended levels | <3 | >0.8 | <0.10 | >0.8 | >0.8 | >0.8 |
| Estimate values for hypothesis model | 2.227 | 0.904 | 0.057 | 0.944 | 0.904 | 0.937 |

**Table S3.** Hypothesis testing results.

| Hypothesis | | Estimate | S.E. | C.R. | P | Testing Results |
|---|---|---|---|---|---|---|
| H1 | ecological environment←management efficiency | 0.517 | 0.057 | 9.214 | *** | Adoption |
| H2 | livelihood assets←management efficiency | 0.488 | 0.05 | 8.785 | *** | Adoption |
| H3 | high-quality ecological products←ecological environment | 0.193 | 0.064 | 3.32 | *** | Adoption |
| H4 | high-quality ecological products←livelihood assets | 0.107 | 0.068 | 1.933 | 0.053 | Rejection |
| H5 | high-quality ecological products←management efficiency | 0.423 | 0.075 | 6.318 | *** | Adoption |
| H6 | social order←management efficiency | 0.644 | 0.073 | 10.827 | *** | Adoption |
| H7 | satisfaction ←high-quality ecological products | 0.268 | 0.056 | 4.611 | *** | Adoption |
| H8 | satisfaction ←ecological environment | 0.163 | 0.057 | 3.022 | 0.003 | Rejection |
| H9 | satisfaction ←livelihood assets | 0.027 | 0.06 | 0.543 | 0.587 | Rejection |
| H10 | satisfaction ←management efficiency | 0.291 | 0.085 | 3.695 | *** | Adoption |
| H11 | satisfaction ←social order | 0.215 | 0.056 | 3.359 | *** | Adoption |

Estimate = Standardized regression weights; S.E. = standardized error; C.R. = critical ratio (>[1.96]). Note: *** $p < 0.001$.
